# Suggestions that there is no visual binding problem ignore evidence

**DOI:** 10.1177/03010066261479194

**Published:** 2026-09-02

**Authors:** Derek H. Arnold

**Affiliations:** 1 School of Psychology, 1974 The University of Queensland, Brisbane, Australia

**Keywords:** visual binding, colour, motion perception lab, school of psychology, The University of Queensland, Australia

## Abstract

Scholte & de Hann (2025) have recently argued that there is no need to integrate information about different types of visual feature – the so-called visual binding problem. Here I argue that they are mistaken, and have ignored key evidence.

Scholte & de Hann (2025) have argued that there is no need to integrate information about different types of visual feature from across functionally specialised regions of cortex - the classical visual binding problem (Livingstone & Hubel, 1988; Zeki, 1978). These authors have argued on the basis that this is not a computational feature of deep neural networks, and they have reiterated seminal arguments that visual neurons tend to jointly encode for multiple features that co-exist in nature (Lennie, 1998). This and similar arguments (e.g., di Lollo, 2012) have made no attempt to explain key evidence that seems to substantiate the existence of a visual binding problem.

The functional architecture of deep language models may be largely irrelevant to human vision. These models do not face the same constraints as biological systems, and while they can perform complex visual tasks, they fail at others that are simple for humans (Campbell et al., 2025; Tangtartharakul & Storrs, 2026). It is likely that these models have a distinct computational architecture to human vision.

More substantive arguments against the visual binding problem (Livingstone & Hubel, 1988; Zeki, 1978) relate to how visual neurons jointly encode for multiple visual features that co-occur in nature (di Lollo, 2012; Lennie, 1998; Scholte & de Hann, 2025). This could mean that there is no need to integrate the products of initially segregated analyses, specialised for different features (like colour and movement) - as envisaged by classical descriptions of the visual binding problem (Livingstone & Hubel, 1988; Zeki, 1978). Such arguments led to the discoveries that the integration of information about spatial form (Nishida, 2004; see Supplemental Movie #1) and colour (Nishida et al., 2007) can be direction-tuned - refuting long-held assumptions that these types of analysis were funcitonally segregated. However, the clearest evidence for a visual binding process would seem to be examples where binding operations are systematically erroneous.

In Steady-State misbinding of colour and motion (Wu et al., 2004), a central stimulus region can contain red dots drifting downward and green dots upward. In the periphery, these contingencies are reversed. When people fixate near the centre of the stimulus, they can have an illusory impression that *all* red dots are coherently drifting upward (see Figure 1 and Supplemental Movie #2). This is an example where the wrong colours and motion directions are perceptually bound in the periphery of vision, informed by contingencies in central vision.

**Figure 1.**
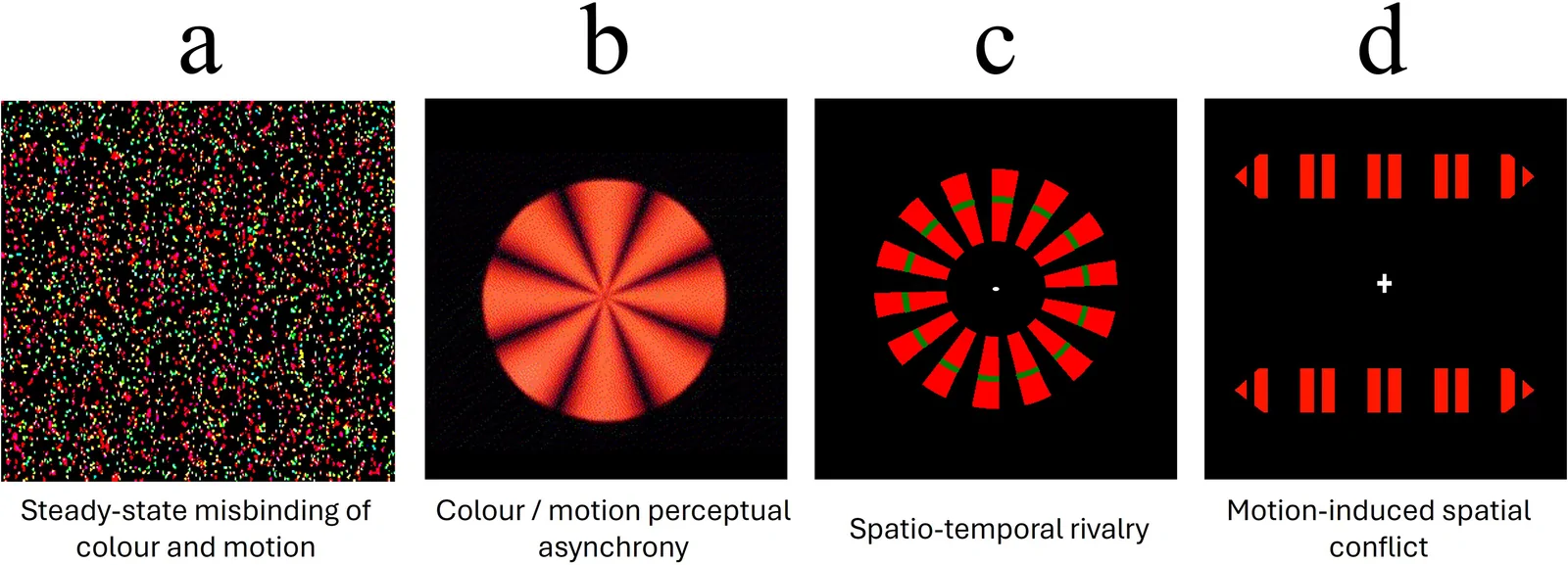
Graphics depicting four visual phenomena that demonstrate failures of visual binding.

In Colour / Motion perceptual asynchrony, people perceive colours as co-occurring with *earlier* periods of movement (Arnold et al., 2001; Moutoussis & Zeki, 1997). In Supplemental Movie #3, there is no physical correspondence between either direction of rotation and either stimulus colour (red or green), as colour changes happen at the mid-point of each rotation period. But people report the stimulus appears to be primarily rotating clockwise while green. The causes of this illusion are debated (Arnold et al., 2001; Nishida & Johnston, 2002; Moutoussis & Zeki, 1997), but common to all accounts are proposals regarding how colours become perceptually bound with earlier periods of motion.

Spatio-temporal rivalry (Arnold et al., 2010) can be induced by a stimulus consisting of a predominantly red windmill pattern, with thin green arcs at the mid-point of each windmill arm (see Supplemental Movie #4). When this pattern is spun rapidly, two illusions can be seen. Coinciding with the locations of the spinning green arcs, an illusory static green ring can be seen. Sections or the entirety of the illusory green ring can then intermittently disappear, to be replaced in perception by illusory impressions of entirely red rotating windmill arms. Rather than arriving at a coherent perceptual outcome, this stimulus pits the perceptual products of components of the human visual system against one another, resulting in a form of perceptual rivalry. This perceptual fragmentation and competition would not happen if components of the human visual system were not specialised to produce different types of experience.

Motion-induced spatial conflict (Arnold et al., 2003) can occur when moving borders defined primarily by colour (the green bars in Supplemental Movie #5) are positioned near moving borders defined by brightness differences (the edges of the red squares). Instead of the two types of moving border seeming to move coherently together, the border defined by differences in colour can seem to shake (or flicker) as it moves. This would seem to be another failure of perceptual binding, where human vision fails to make different types of motion cohere in perception.

Suggestions that there is no visual binding problem (e.g., di Lollo, 2012; Scholte & de Haan, 2025) tend to ignore or dismiss the evidence of erroneous perceptual bindings of colour and motion. Scholte & de Haan (2025) also ignore their own evidence. They were involved in a study of a stroke cohort (Lugtmeijer et. al., 2025), where they found evidence linking selective deficits of motion perception to focal damage of the right temporo-parietal junction, and to the right lateral occipital cortex. These brain regions are involved in the integration of visual information (e.g., Jitsuishi et al., 2020). So this evidence linking instances of impaired motion perception to focal brain damage refutes the authors’ conclusions that there is no such evidence (Lugtmeijer et. al., 2025; Scholte & de Hann. 2025).

Questions about visual binding are among the most challenging in science, as they explicitly relate to phenomenal experience (see Percy & Agarwal, 2026). We are asking why things look as they do (Koffka, 1922)? Lennie (1998) made a seminal contribution to these discussions. He challenged us to consider the perceptual consequence of visual neurons that are jointly responsive to multiple visual features. Evidence in support of the perceptual relevance of these neurons followed (e.g., Nishida, 2004; Nishida et al., 2007). This and other evidence suggest that the visual binding problem might not be the same problem as was once envisaged (Livingstone & Hubel, 1988; Zeki, 1978). However, the claim that there is no visual binding problem seems to be refuted by instances where the wrong combinations of colour and of movement are bound in perception (e.g., Arnold et al., 2003; Arnold et al., 2001; Arnold et al., 2010; Moutoussis & Zeki, 1997; Nishida & Johnston, 2002; Wu et al., 2004). Ignoring the phenomenal evidence that seems to substantiate some form of visual binding problem will not make that problem go away. It will just mean that models and theoretical accounts of human vision are only partially informed.

## Supplemental Material

**Figure:** Video 1.

**Figure:** Video 2.

sj-gif-1-pec-10.1177_03010066261479194 - Supplemental material for Suggestions that there is no visual binding problem ignore evidence Supplemental material, sj-gif-1-pec-10.1177_03010066261479194 for Suggestions that there is no visual binding problem ignore evidence by Derek H. Arnold in Perception

**Figure:** Video 3.

**Figure:** Video 4.
